# NOTE: non-parametric oversampling technique for explainable credit scoring

**DOI:** 10.1038/s41598-024-78055-5

**Published:** 2024-10-30

**Authors:** Seongil Han, Haemin Jung, Paul D. Yoo, Alessandro Provetti, Andrea Cali

**Affiliations:** 1 School of Computing & Mathematical Sciences, University of London, Birkbeck College, London, UK; 2 Department of Industrial & Management Engineering, Korea National University of Transportation, Chungju, South Korea

**Keywords:** Conditional Wasserstein generative adversarial networks, Stacked autoencoder, Explainable AI, Imbalanced class, Oversampling, Credit scoring, Computer science, Information technology

## Abstract

Credit scoring models are critical for financial institutions to assess borrower risk and maintain profitability. Although machine learning models have improved credit scoring accuracy, imbalanced class distributions remain a major challenge. The widely used Synthetic Minority Oversampling TEchnique (SMOTE) struggles with high-dimensional, non-linear data and may introduce noise through class overlap. Generative Adversarial Networks (GANs) have emerged as an alternative, offering the ability to model complex data distributions. Conditional Wasserstein GANs (cWGANs) have shown promise in handling both numerical and categorical features in credit scoring datasets. However, research on extracting latent features from non-linear data and improving model explainability remains limited. To address these challenges, this paper introduces the Non-parametric Oversampling Technique for Explainable credit scoring (NOTE). The NOTE offers a unified approach that integrates a Non-parametric Stacked Autoencoder (NSA) for capturing non-linear latent features, cWGAN for oversampling the minority class, and a classification process designed to enhance explainability. The experimental results demonstrate that NOTE surpasses state-of-the-art oversampling techniques by improving classification accuracy and model stability, particularly in non-linear and imbalanced credit scoring datasets, while also enhancing the explainability of the results.

## Introduction

Credit risk is defined as the potential for loss arising from the creditworthiness of applicants^1^. This implies that credit risk can significantly impact non-performing financial obligations, which are closely associated with bankruptcy. Given its potential to affect the sustainability of financial institutions, accurately managing credit risk through precise credit scoring is of paramount importance^2^.

Logistic regression (LR) has been the most commonly employed method for credit scoring evaluation and is widely regarded as the standard in this field. As a parametric model, LR models are both explainable and interpretable. They require less computational resources and time, as well as smaller training datasets. Parametric models can be expressed as functions that relate the target feature to input features in a linear and relational manner. However, the primary limitation of parametric models, including LR, is their constrained predictive power, which is considered their main drawback^3^.

Conversely, tree-based models such as random forest (RF) and gradient boosting (GB) are non-parametric. Non-parametric models are trained with minimal functional assumptions during the learning process from the dataset^3^. This attribute endows these models with greater flexibility, resulting in superior predictive performance compared to logistic regression (LR). However, while parametric models like LR are easily interpretable, the predictions generated by non-parametric tree-based models are more challenging to explain.

Today, significant effort is being devoted to applying state-of-the-art machine learning (ML) technologies to credit scoring. However, two main issues persist^4^, namely, imbalanced class distribution in datasets and model explainability. In classification problems, maintaining a balanced dataset is crucial as it enhances the learning process. ML models typically assume that datasets have a similar number of observations in each class^5^. However, in real-life applications of credit scoring, datasets are often imbalanced. Models trained on imbalanced datasets tend to predict the most common class to maximize overall classification accuracy, which can lead to label misclassification^6,7^. Imbalanced class distribution refers to a scenario where the ratio of observations in each class is uneven and skewed toward one class^8^. Most methods addressing the problem of imbalanced class distribution aim to improve classification accuracy for the minority class.

To address the problem of class imbalance at the data level, re-sampling methods have been widely employed^9^. These methods balance the classes either by reducing the number of majority class instances or by increasing the number of minority class instances. A typical oversampling technique is the Synthetic Minority Over-sampling TEchnique (SMOTE)^10^. Oversampling techniques leverage all available information in the dataset, whereas undersampling techniques discard portions of the available data^11^. However, a potential issue with oversampling is that it can generate overlapping data, which may be perceived as additional random noise^12^. Models trained on datasets containing duplicated data are prone to overfitting.

To address the limitations of conventional oversampling techniques, a Generative Adversarial Networks (GAN)-based oversampling approach has recently been proposed. GANs learn the overall distribution of the minority class and generate new samples that closely resemble real data from this class. Consequently, the generated distribution can capture latent characteristics present in the original dataset. Furthermore, GANs have been reported to mitigate issues such as overfitting, class overlapping, and the introduction of additional noise, which are common limitations of traditional oversampling methods.

This study extends the GAN-based oversampling technique and proposes a novel approach named NOTE (Non-parametric Oversampling Technique for Explainable Credit Scoring). In addition to unsupervised generative learning, NOTE effectively extracts latent features using a Non-parametric Stacked Autoencoder (NSA) to capture complex and non-linear patterns. Furthermore, it incorporates eXplainable Artificial Intelligence (XAI) methodologies, employing SHAP (SHapley Additive exPlanations) as suggested by Lundberg and Lee^13^, to elucidate the classification process.

The key contribution of this study consists of the following:

- To demonstrate the effectiveness of latent feature extraction using the Non-parametric Stacked Autoencoder (NSA), comparing it to the denoising method via randomized Singular Value Decomposition (rSVD) within non-linear credit scoring datasets
- To present the advancements of conditional Wasserstein GAN (cWGAN) in addressing mode collapse during GAN training, evaluating its suitability, stability, and superiority in generating the minority class in imbalanced datasets relative to benchmarks such as ADS-GAN^14^ and DeepSMOTE^15^
- To propose the architecture for a non-parametric model specifically designed for non-linear and imbalanced datasets, offering a robust solution
- To introduce new benchmark results that exceed the performance of state-of-the-art models^12^ on the Home Equity (HE) and Give Me Some Credit (GMSC) datasets within the credit scoring literature
- To ensure the practical applicability of the model in credit scoring by improving its transparency through eXplainable Artificial Intelligence (XAI)

We propose that the NOTE framework has the potential to surpass existing benchmarks by offering enhanced performance and improved interpretability.

The remainder of this paper is as follows: Section “Related work” provides a review of related studies and identifies gaps in current methodologies. Section “Methods” outlines the proposed NOTE framework and its novel features. Section “Results” presents the results, comparing the performance of NOTE with benchmarks from recent research. Finally, Section “Discussion” discusses the key findings, limitations, and potential directions for future work.

## Related work

This section reviews related studies on GAN-based oversampling and the synthesis of tabular data containing both numerical and categorical features using GANs.

### GAN-based oversampling

Traditional oversampling methods, such as SMOTE, often rely on duplicating and interpolating existing data, which can lead to overfitting and biased model performance by replicating patterns from the original dataset. This is particularly problematic in structured data applications like credit scoring, where the model’s ability to generalize is crucial for predicting outcomes on unseen data. In contrast, GAN-based oversampling techniques provide a more sophisticated approach by learning the underlying data distribution and generating synthetic samples that mimic real-world data. By generating more diverse and realistic synthetic samples, GAN-based techniques mitigate the risk of overfitting, thus addressing the limitations posed by traditional methods like SMOTE.

Leveraging this advantage, GANs have been applied to various domains such as image processing, video analysis, and computer vision, demonstrating strong performance with unstructured data^16,17^. Recently, extensive studies based on GANs have emerged to address issues of class imbalance and missing values in structured or tabular data.

Recent literature has highlighted the effectiveness of GANs across multiple domains, particularly in credit scoring and imbalanced classification. For instance, Fiore et al.^18^ demonstrated the superior performance of vanilla GAN in fraud detection within credit card transactions, showing that it outperformed SMOTE in terms of classification effectiveness. Similarly, Douzas and Bacao et al.^19^ introduced conditional GAN (cGAN) to oversample minority classes, outperforming several SMOTE variants on various imbalanced datasets. Although these studies showed considerable improvement in imbalanced learning, their primary focus was on datasets with only numerical features, neglecting the challenges associated with generating categorical features.

Addressing this limitation, Seo et al.^20^ proposed a meta-learning methodology integrated with GANs to prevent overfitting-a common issue in traditional oversampling techniques. Their approach was validated on an imbalanced loan dataset, where GANs demonstrated superior performance compared to SMOTE in handling minority class predictions. In a similar vein, Xu and Veeramachaneni et al.^21^ introduced tabular GAN (tGAN), which synthesized both continuous and discrete features, outperforming other generative models in structured tabular data. Additionally, Son et al. et al.^22^ proposed borderline-cGAN (bcGAN) to better define boundaries between majority and minority classes, further enhancing the classification performance compared to SMOTE in highly imbalanced datasets.

Despite these developments, many studies have fallen short in generating both categorical and numerical features, a critical requirement in credit scoring datasets that typically comprise a mixture of feature types. To address this gap, Engelmann and Lessmann^12^ introduced conditional Wasserstein GAN (cWGAN), a technique capable of generating synthetic samples for both categorical and numerical features. Their research demonstrated that cWGAN effectively balanced class distributions in non-linear credit scoring datasets, significantly improving classification performance over traditional methods such as SMOTE.

Additionally, a more recent development in 2023 introduced the Adaptive-Robust Data Balancing Oversampling TEchnique (A-RDBOTE model)^23^, specifically designed for credit scoring applications. This model generates robust synthetic samples and improves upon conventional techniques by tackling the issues of overfitting and bias associated with traditional oversampling techniques. By leveraging the flexibility of GAN-based approaches, A-RDBOTE showcases the continuous advancements being made in the realm of credit scoring and imbalanced classification.

Zhu et al.^24^ further extended the application of GAN-based methods in structured data by developing a hybrid GAN approach to handle imbalanced customer classification. Their findings revealed that GANs were more effective at generating realistic synthetic data for both numerical and categorical features, significantly improving the model’s performance in handling class imbalances. This underscores the growing utility of GAN-based techniques in oversampling for structured datasets, offering more robust solutions than traditional methods.

In conclusion, while the literature underscores the progress made in GAN-based oversampling methods, there remains a need for continued research on generating both categorical and numerical features, particularly in structured datasets like those used in credit scoring. The advancements in models such as cWGAN and A-RDBOTE reflect the ongoing efforts to address these gaps, providing more comprehensive solutions for imbalanced learning in the credit scoring domain. By enhancing dataset balance and improving classification accuracy, these models contribute meaningfully to overcoming the limitations inherent in traditional oversampling techniques.

### Generating tabular data by GAN

Generative Adversarial Networks (GANs) have demonstrated considerable efficacy in generating synthetic data, particularly in the context of imbalanced datasets. Their capacity to learn and replicate complex data distributions renders them a promising approach for the generation of synthetic tabular data. However, despite their significant potential, GANs face several challenges when applied to tabular data, which typically comprises a mix of categorical and numerical features, intricate interdependencies, and varying degrees of sparsity.

One of the most critical challenges in training GANs is mode collapse, where the generator fails to capture the full diversity of the data distribution^25,26^. In the context of tabular data, mode collapse can result in the generator producing a limited range of outputs that do not adequately reflect the various categories or numerical ranges present in the original dataset. For instance, in a credit scoring dataset, mode collapse may lead to the generation of synthetic samples that represent only a subset of potential credit scores or borrower profiles, thereby diminishing the utility of the generated data.

Mode collapse is often identified when the generator outputs overly similar results or when the generator’s loss curve becomes flat, indicating a failure to effectively learn the data distribution. To mitigate mode collapse, several techniques, including minibatch discrimination and spectral normalization, have been introduced^25,26^. Minibatch discrimination encourages the generator to produce a greater diversity of outputs by allowing the discriminator to assess patterns across a batch of data, rather than evaluating individual samples independently. Spectral normalization, on the other hand, stabilizes GAN training by controlling the Lipschitz constant of the discriminator, ensuring that the generator learns meaningful features while avoiding mode collapse. These techniques collectively enhance the generator’s ability to produce outputs that more accurately reflect the original data distribution^25,26^.

In addition to mode collapse, training instability is another significant challenge in using GANs for generating tabular data^25,26^. Training instability arises from the adversarial nature of GANs, where the generator and discriminator are engaged in a continuous competition to outperform each other. This competitive dynamic can lead to oscillating loss curves, with neither network reaching convergence, ultimately resulting in the production of low-quality synthetic data. In the context of tabular data, this instability may manifest as the generation of unrealistic or nonsensical combinations of features that do not align with the logical or statistical properties of the original data.

Training instability can be further aggravated by factors such as vanishing gradients, where the generator receives insufficient feedback from the discriminator, or by poor initialization and suboptimal hyperparameter settings, which can lead to erratic and unpredictable training processes^25,26^.

Given that tabular datasets for credit scoring typically encompass various data types, including both numerical and categorical features, and recognizing that numerical values can exhibit complex distributions such as multi-modal or thick-tailed patterns, the simultaneous generation of both numerical and categorical data using GANs can be particularly challenging^21^. Specifically, numerical data may need to adhere to certain constraints, such as being within a specific range and positive, e.g., age should be between 1 and 100 and should be an integer. For categorical data, it may be nominal, ordinal, or a combination of both.

Consequently, numerous studies have been proposed to generate synthetic tabular data with both numerical and categorical features using variants of GAN. These studies focus on addressing the challenges of generating diverse types and characteristics of data simultaneously and overcoming the limitations of the original GAN, such as mode collapse when modeling categorical values and complex numerical distributions like multi-modal datasets^27^.

Choi *et al*.^28^ proposed Medical GAN (MedGAN) to generate categorical synthetic data for binary columns in patient diagnosis records within the healthcare domain. This study addressed the limitation of privacy risk by combining an autoencoder with GAN. Xu *et al*.^29^ developed conditional tabular GAN (ctGAN) to model multi-categorical columns using an architecture that incorporates one-hot encodings, a softmax activation function, and Wasserstein distance for loss updating, building on their previous work with tGAN^21^. Their study demonstrated improved generative performance for categorical features compared to the earlier tGAN model.

Therefore, these studies demonstrated that modeling tabular synthetic data for complex numerical distributions and multi-categorical data simultaneously depends heavily on the GAN architecture.

Although previous studies have demonstrated notable success in various domains, they are accompanied by substantial challenges. In addressing these challenges, the introduction of techniques like the Wasserstein GAN (WGAN) has been pivotal^30^. WGAN replaces the traditional GAN loss function with the Wasserstein distance, thereby promoting a more stable gradient flow, which aids the generator in producing more realistic data. This modification to the loss function results in more stable training compared to conventional GAN models.

However, the original WGAN implementation enforced a Lipschitz constraint on the discriminator by using weight clipping, a method that often caused difficulties during training, such as the production of low-quality samples or failure to converge. To overcome these issues, Gulrajani et al.^31^ proposed the use of WGAN with Gradient Penalty (WGANGP). This approach penalizes the norm of the gradient of the discriminator concerning its inputs, ensuring that the Lipschitz continuity is maintained. As a result, the gradient is prevented from becoming excessively large or small, which could destabilize the training process^25,26^, thereby enabling smoother and more stable training. Due to its robustness and capacity to generalize across various models, this method has become a widely adopted improvement in GAN training. The Conditional Wasserstein GAN (cWGAN) further refines this methodology by conditioning both the generator and discriminator on auxiliary information, such as class labels. This conditioning enhances the generation of synthetic data that aligns with specific characteristics of tabular datasets.

Despite these advancements, precise hyperparameter tuning remains indispensable for ensuring the stability and effectiveness of the training process. Nevertheless, when these challenges are adequately addressed, GANs demonstrate significant potential in generating high-quality, diverse, and representative tabular data. The effective integration of advanced methodologies to mitigate issues such as mode collapse and training instability is crucial for fully harnessing the capabilities of GANs, particularly in critical applications like credit scoring, where the accuracy and reliability of synthetic data are paramount.

Consequently, several enhancements to GAN architecture, including modifications to its structure and loss function, have been introduced to address these challenges. In this study, we apply these advancements in GAN technology to oversample the minority class, aiming to balance the credit scoring dataset and improve model performance in this domain.

## Methods

### The framework of NOTE

The NOTE framework comprises four sequential stages, outlined as follows:

1. Collecting credit scoring datasets
2. Extracting latent representations using the non-parametric stacked autoencoder (NSA) and merging these with the original dataset
3. Oversampling the minority class (defaulted credit samples) using conditional Wasserstein GAN (cWGAN)
4. Predicting classifications and elucidating the model using TreeExplainer and LinearExplainer

These stages must be executed in sequence to achieve the desired levels of effectiveness.

The Home Equity (HE) dataset, referenced by Baesens et al.^32^, includes characteristics and delinquency data for mortgage applicants, derived from prior research within the credit scoring literature. The dataset consists of 5,960 samples, of which 4,771 are labeled as non-defaulted and 1,189 as defaulted cases. In this dataset, the target feature ‘BAD’ serves as the binary class label, where a value of 1 indicates a defaulted credit case, and a value of 0 indicates that the applicant has met their financial obligations. This dataset was selected to evaluate the robustness of the NOTE framework due to its non-linear characteristics, as previously noted by Engelmann and Lessmann^12^.

**Figure 1:**
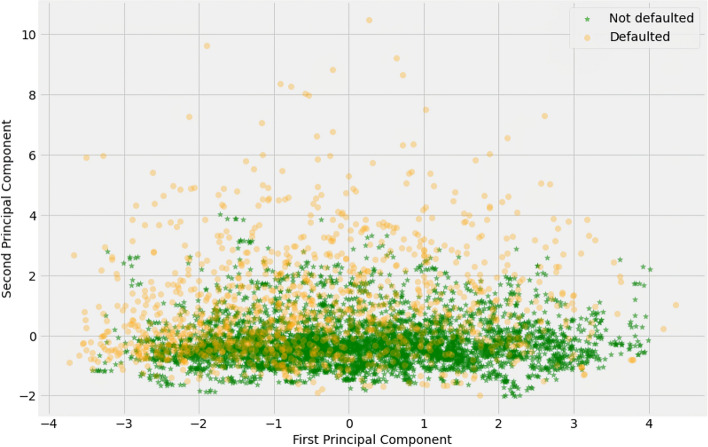
Visualization of non-linear and imbalanced class distribution on HE dataset using principal component analysis.

**Table 1:** Features on HE and GMSC datasets used in NOTE.

Feature	Description of HE	Type
$\textbf{BAD}^{*}$	Applicant paid loan, or applicant defaulted on loan or seriously delinquent	N (Not defaulted = 0) / Y (Defaulted = 1)
LOAN	Amount of the loan request	Integer
MORTDUE	Amount due on existing mortgage	Integer
VALUE	Value of current property	Integer
REASON	DebtCon = debt consolidation; HomeImp = home improvement	Category
JOB	Six occupational categories (Other, ProfExe, Office, Sales, Mgr, Self)	Category
YOJ	Years at present job	Integer
DEROG	Number of major derogatory reports	Integer
DELINQ	Number of delinquent credit lines	Integer
CLAGE	Age of oldest credit line in months	Real
NINQ	Number of recent credit inquiries	Integer
CLNO	Number of credit lines	Integer
DEBTINC	Debt-to-income ratio	Real
**Feature**	$$\textbf{Description of GMSC}^{***}$$	**Type**
$$\textbf{SeriousDlqin2yrs}^{**}$$	Applicant experienced 90 days past due delinquency or worse	N (Not defaulted = 0) / Y (Defaulted = 1)
RevolvingUtilizationOfUnsecuredLines	Total balance on credit cards and personal lines of credit	Percentage
Age	Age of in years	Integer
NumberOfTime30–59DaysPastDueNotWorse	Number of times applicant has been 30–59 days past due but no worse in the last 2 years	Integer
DebtRatio	Monthly debt payments, alimony,living costs divided by monthly gross income	Percentage
MonthlyIncome	Monthly income	Real
CombinedCreditLoans	Number of open loans and lines of credit, including real estate and home equity	Category
CombinedDefaulted	Number of times an applicant has been 30–89 days or over 90 days past due	Category
NumberOfDependents	Number of dependents in family excluding themselves (spouse, children etc.)	Category

*BAD and **SeriousDlqin2yrs are features for class label.

***The GMSC dataset has been feature-engineered to derive categorical features from the original variables.

The HE dataset also presents a significant class imbalance, with an imbalance ratio (IR) of 4.012, calculated by dividing the number of majority class instances by the number of minority class instances. Table 1 provides an overview of the HE dataset’s features, while Fig. 1 illustrates the distribution of imbalanced classes within the dataset using Principal Component Analysis (PCA) with two principal components. The plot reveals a notable class imbalance, as the “Not Defaulted” class (green points) significantly outnumbers the “Defaulted” class (yellow points), reflecting the typical distribution of credit data. Additionally, the figure highlights the complexity of the relationship between the two classes. Despite dimensionality reduction, the two classes remain highly intertwined, with substantial overlap between the points representing defaulted and non-defaulted instances. This overlap suggests that the classes are not linearly separable in this space, indicating that the distinguishing characteristics between them are intricate and non-linear. As a result, more sophisticated modeling approaches are required to effectively separate the classes and address the imbalanced nature of the dataset.

Similarly, the Give Me Some Credit (GMSC) dataset^12^, sourced from the Kaggle competition “Give Me Some Credit,” contains demographic information, payment behavior, and delinquency records, and is widely recognized as a benchmark dataset in credit scoring research. The GMSC dataset consists of 150,000 samples, of which approximately 140,000 are labeled as non-defaulted and 10,000 as defaulted. The target feature ‘SeriousDlqin2yrs’ serves as a binary class label, with a value of 1 indicating defaulted cases and a value of 0 indicating non-defaulted cases. The GMSC dataset was selected to further validate the generalizability of non-parametric models in non-linear credit scoring contexts^12^.

This dataset also exhibits a significant class imbalance, with the minority class constituting only 6.684% of the total, resulting in an IR of 13.961. The GMSC dataset contains 10 features, excluding the target variable, all of which are highly interpretable within the context of credit scoring. A detailed description of the HE and GMSC datasets is provided in Table 1. These datasets were chosen for their established non-linear and imbalanced characteristics and benchmark status in recent credit scoring studies, offering a robust foundation for evaluating the effectiveness of the NOTE framework.

**Figure 2:**
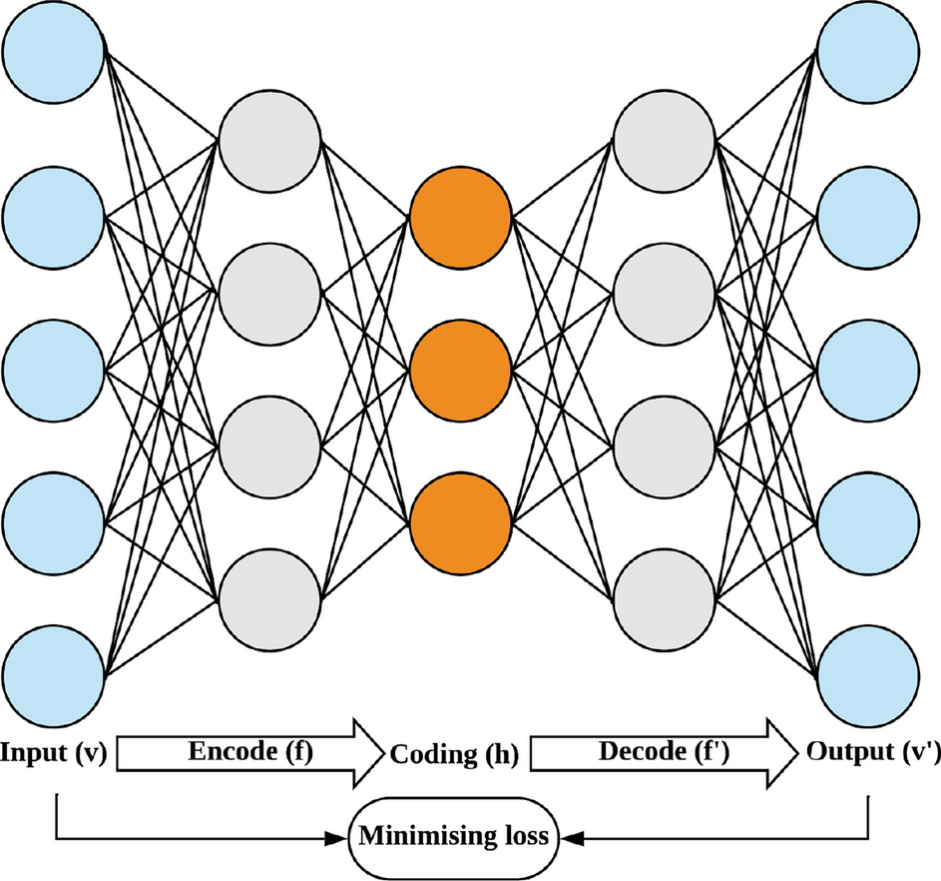
The non-parametric stacked autoencoder (NSA), where codings are latent features of original dataset.

### Feature generation by NSA

By extracting latent representations, non-linear information is obtained from the dataset. To address the limitations of PCA, which captures only linear characteristics for feature extraction, an autoencoder can be recommended and applied to extract non-linear patterns when the dataset exhibits strong non-linearity and complexity.

A neural network is employed to perform non-linear transformations and generate latent characteristics for learning data representations^33^. This process can be conducted using a non-parametric stacked autoencoder (NSA), an unsupervised learning method comprising an encoder (which encodes input data to create latent representations or codings) and a decoder (which decodes the latent representations or codings to reconstruct the input)^34^. Figure 2 illustrates the structure of the NSA. The NSA is trained to minimize the loss, i.e., minimizing the reconstruction error. The loss function *L* can be expressed as follows:

1 $$\begin{aligned} L(v, v') = \frac{1}{n} \sum _{v_{k} \in D_{train}}\parallel v-v'\parallel ^{2} \end{aligned}$$,

where $D_{train}=\{(v_{k})\}_{k=1}^{n}$ is a training set.

Thus, the codings can be considered as the extracted outcomes from the representation learning process.

Following this approach, the latent vectors, denoted as coding *h* in Fig. 2 and extracted via the Non-parametric Stacked Autoencoder (NSA), are concatenated with the original dataset. As a result, the dimensionality of the feature space increases from the initial number of features to the sum of the original features and the latent vectors, reflecting the inclusion of the extracted latent features as describe in Fig. 5. This augmentation of the dataset through the incorporation of latent representations enhances the model’s capacity to identify and model complex relationships and patterns within the data, leading to improved model performance.

Furthermore, Engelmann and Lessmann^12^ demonstrated that the oversampling technique using cWGAN exhibited optimal classification performance in non-linear datasets when combined with tree-based models such as Random Forest (RF) and Gradient Boosting (GB). Consequently, their findings suggest that promising performance can be expected if oversampling by cWGAN is integrated with NSA for strongly non-linear datasets, and the resulting dataset with latent vectors is subsequently paired with tree-based machine learning algorithms, such as Extra Trees (ET), RF, and GB.

### cWGAN-based oversampling

Following feature generation by NSA, NOTE employs cWGAN-based oversampling to balance the classes within the imbalanced dataset. Since an imbalanced dataset can introduce bias during model training, the minority class is oversampled to match the number of instances in the majority class prior to the model training phase.

**Figure 3:**
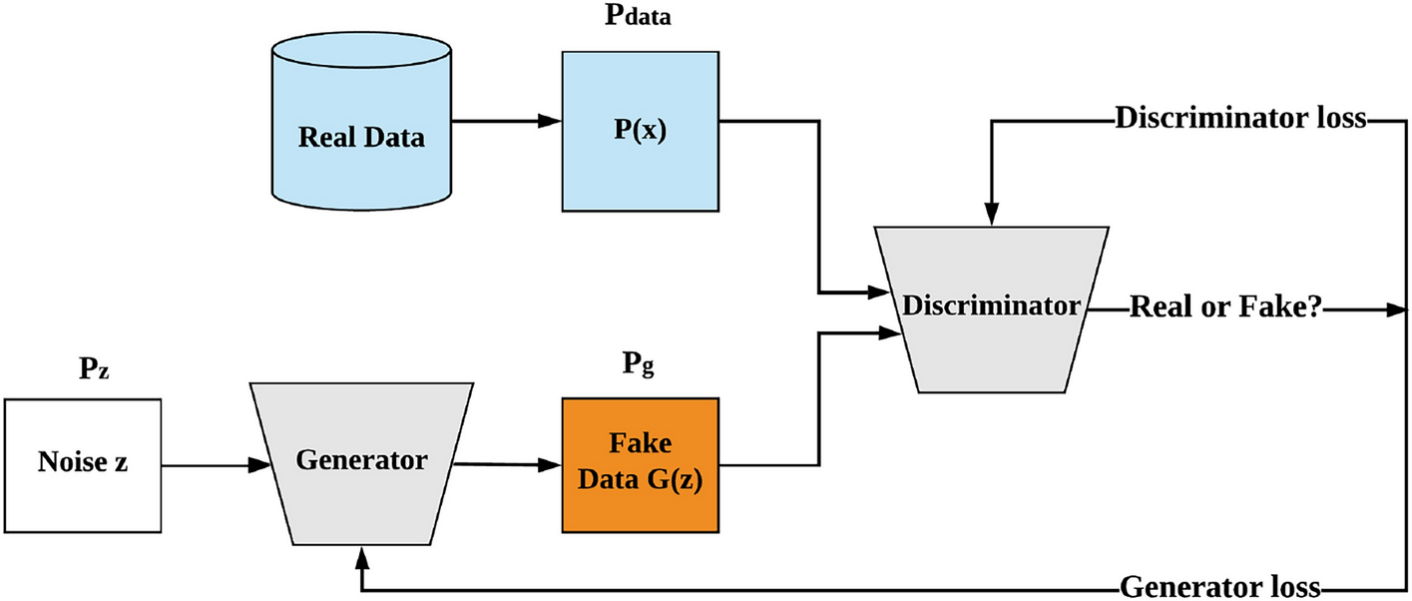
The structure of GAN-based generation for synthetic data.

Generative adversarial networks (GANs)^35^ represent an architectural framework for learning generative models through an adversarial process, comprising two distinct models. The first model, the generator (*G*), produces artificial samples that aim to replicate the distribution of real data. The second model, the discriminator (*D*), endeavors to differentiate between real data and the synthetic samples produced by the generator (*G*)^12^.

The generator (*G*) takes a latent vector or noise (*z*) from a noise distribution ($P_{z}$) as input and maps the noise (*z*) into the data space ($\mathcal {X}$) to generate synthetic data. As previously discussed, the two models, *G* and *D*, are trained simultaneously: *G* is trained to produce synthetic or fake samples that closely resemble real data to deceive *D*, while *D* is trained to distinguish between real samples and the generated fake samples. This process is referred to as a two-player minimax game, and it can be expressed with the following loss function:

2 $$\begin{aligned} \min _{G} \max _{D} V(G, D) = \mathbb {E}_{x \sim P_{data}(x)}[\log D(x)] + \mathbb {E}_{z \sim P_{z}(z)}[\log (1 - D(G(z)))] \end{aligned}$$,

where $P_{data}$ represents the distribution of real data, $P_{z}$ denotes the distribution of the latent vector or noise, and *D*(*x*) is the probability that *x* follows the real data distribution $P_{{data}}$ rather than $P_{g}$, which signifies the distribution of data generated by the generator *G*.

Figure 3 illustrates the structure of GAN-based generation. The objective of the generator (*G*) is achieved when the generated distribution $P_{g}$ is equal to the real distribution $P_{data}$ as demonstrated by^35^. This is equivalent to minimizing the Jensen-Shannon Divergence (JSD), which measures the similarity between the two distributions^12^.

**Figure 4:**
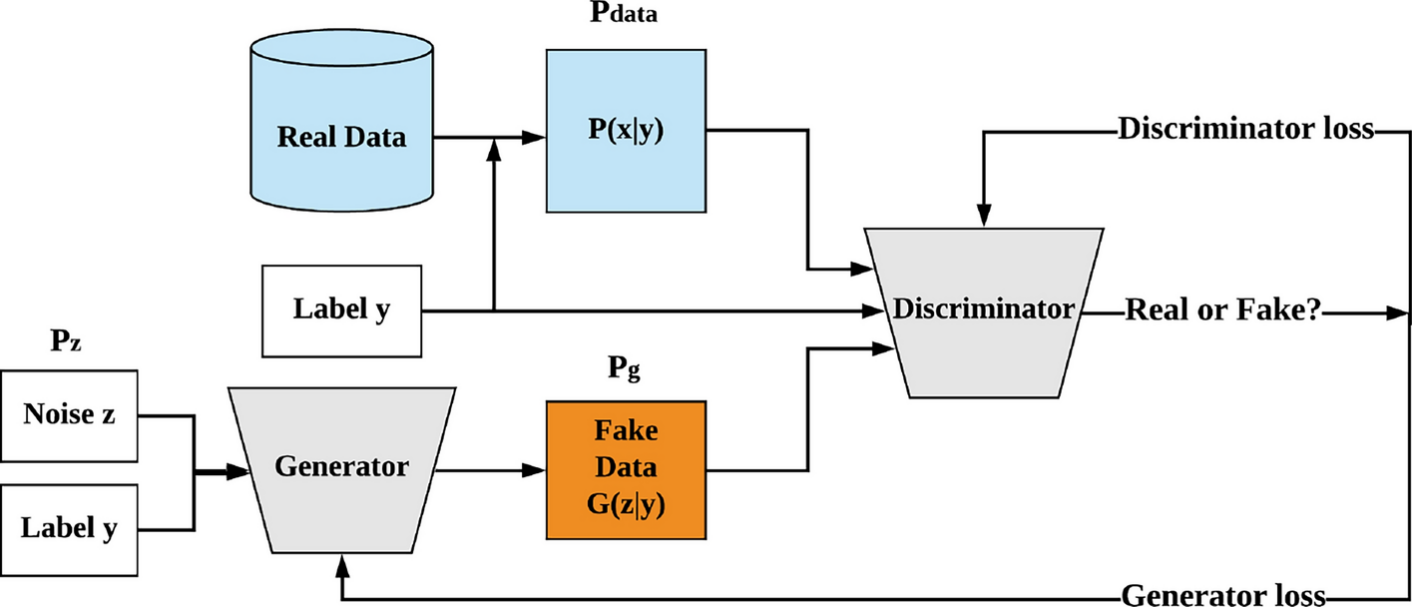
The structure of cGAN-based generation for synthetic data.

Since it is not possible to impose restrictions or conditions on synthetic samples when generating data with vanilla GAN, conditional GAN (cGAN)^36^, a variant of vanilla GAN, was proposed to address this limitation.

Although cGAN follows a learning process similar to that of GAN, there is a key difference in the input variable of the generator *G*^36^. In cGAN, the generator *G* takes both a condition *y* and noise *z* as inputs and maps them into the data space $\mathcal {X}$ to generate new data. Subsequently, the two models, *G* and *D*, are trained together in the same manner as in GAN. Figure 4 illustrates the structure of cGAN-based generation.

The condition *y* can be regarded as additional information that both the generator *G* and the discriminator *D* consider when generating and discriminating fake data. This allows the generated samples to belong to a specific class while enabling *G* to incorporate the class label. In other words, imposing this condition can stabilize the training process^12^. The loss function for cGAN can be expressed as follows:

3 $$\begin{aligned} \min _{G} \max _{D} V(G, D) = \mathbb {E}_{x \sim P_{\text {data}}(x|y)}[\log D(x|y)] + \mathbb {E}_{z \sim P_{z}(z)}[\log (1 - D(G(z|y)))] \end{aligned}$$

In the field of machine learning, one of the most crucial procedures is the process of learning the probability distribution of data. Therefore, learning the joint probability distribution of data is essential for developing a generative model. This process is equivalent to maximizing the likelihood estimation, which corresponds to minimizing the Kullback-Leibler Divergence (KLD). This can be expressed as follows:

4 $$\begin{aligned} \arg \max _{\theta } \sum _{i=1}^{n} \log P_{\theta }(x_{i}) = \int _{x} P_{r}(x) \log P_{\theta }(x) \, dx = \arg \min _{\theta } \text {KL}(P_{r} \parallel P_{\theta }) \end{aligned}$$

Kullback-Leibler Divergence (KLD) quantifies the extent to which one probability distribution approximates or deviates from a reference distribution^37^. This divergence can be represented by Jensen-Shannon Divergence (JSD), which similarly measures the similarity between two distributions^38^. Both KLD and JSD are mathematical methods employed to minimize the discrepancy between two distributions.

According to KLD and JSD, the ideal reference distribution and the approximate distribution should have the same support if the two distributions are similar. The support of a probability distribution is the set of values that the probability variable $P(\cdot )$ can take, and it can be expressed as $\lbrace x | P(x)>0\rbrace$.

However, in real data space, the supports of the two distributions are not the same since meaningful information is typically concentrated in a small portion of the data space compared to the entire data space $\mathcal {X}$. Consequently, it is challenging for the GAN loss function to accurately calculate the distance between the generated distribution $P_{g}$ and the real distribution $P_{data}$. This difficulty can lead to issues such as mode collapse and unstable learning during the training process of both GAN and cGAN^36^.

To overcome the challenges associated with the training processes of GAN and cGAN, the Wasserstein distance was proposed as an alternative to JSD for measuring the distance between two distributions. The Wasserstein distance has been incorporated by replacing the loss functions of both vanilla GAN and conditional GAN (cGAN), resulting in the development of Wasserstein GAN (WGAN) and conditional Wasserstein GAN (cWGAN), respectively^30^.

The Wasserstein distance can be interpreted as the cost of the optimal transport plan required to move the mass of the probability distribution^12^. Both WGAN and cWGAN are trained to minimize this cost within the loss function. This cost can be expressed as follows:

5 $$\begin{aligned} \text {Cost} = \text {mass} \times \text {distance} = \sum _{p \in P}\sum _{q \in Q} \gamma (p,q) \cdot \Vert p - q \Vert ^m = \mathbb {E}_{\gamma (p,q)}(\Vert p - q \Vert ^m) \end{aligned}$$,

where *p* is one of bin in the support of distribution $P_{r}$, *q* is one of bin in the support of distribution $P_{\theta }$, $\gamma (p,q)$ represents the distance between *p* and *q*, i.e. mass, and for any $m\ge 1$, $\parallel \cdot \parallel$ denotes the Euclidean norm on $\mathbb {R}^{n}$.

As discussed earlier, to address challenges such as mode collapse and training instability in GANs, the WGAN was introduced as an alternative to optimizing the Jensen-Shannon Divergence (JSD). The WGAN framework promotes greater training stability by offering a more consistent and informative gradient, even in cases where the generated samples significantly diverge from the true data distribution. However, the implementation of WGAN can still encounter issues such as vanishing or exploding gradients. These issues are mitigated by the WGANGP, which introduces a gradient penalty to ensure that the discriminator adheres to the 1-Lipschitz continuity^31^. This penalty acts as a regularizer, softly enforcing the Lipschitz constraint, thereby maintaining a smooth gradient in the discriminator. This smooth gradient is crucial for guiding the generator effectively in learning the data distribution. Consequently, WGANGP contributes to more stable convergence and addresses mode collapse by penalizing the discriminator’s gradient.

In CGAN, conditioning the generator allows for the generation of outputs that correspond to a specific class, while conditioning the discriminator ensures that the generator cannot disregard the class label. Empirical evidence has demonstrated that this conditioning contributes to a more stable training process^12^.

In conclusion, the Conditional Wasserstein GAN (cWGAN) is employed to mitigate the challenges posed by class imbalance in tabular data, with a particular focus on the domain of credit scoring. The proposed cWGAN-based oversampling method enhances the traditional GAN framework by conditioning both the generator and the discriminator on class labels. This approach is specifically designed to generate synthetic samples that pertain to the minority class, thereby improving the overall balance within the dataset.

The principal components of the cWGAN-based oversampling method are as follows:

- Objective of cWGAN: A cGAN structure is utilized to estimate the conditional distribution of the data, given the class label. The generator is conditioned on the minority class label to explicitly produce minority class samples. The loss function incorporates the WGANGP to ensure stability^31^, alongside an auxiliary classifier (AC) loss, which encourages the generator to generate samples that are not only realistic but also accurately classified according to their respective labels^39^.
- Modeling tabular data: The architecture is specifically designed to manage both numerical and categorical variables. For categorical data, the Gumbel-softmax function is employed to model the output distribution, while numerical data is processed through a scaled, unclipped output, allowing the generator to learn the appropriate range of values. Crosslayers are implemented to model interactions between features, thereby enhancing the generator’s capability to produce realistic data.
- Network architecture: The architecture of both the generator and discriminator is designed to incorporate embedding layers, which enhance the representation of categorical data. Furthermore, the architecture includes parallel crosslayers that facilitate higher-order interactions among features. This sophisticated design aims to generate synthetic data that closely replicates the real data distribution, ensuring the accurate representation of relationships between features.

**Figure 5:**
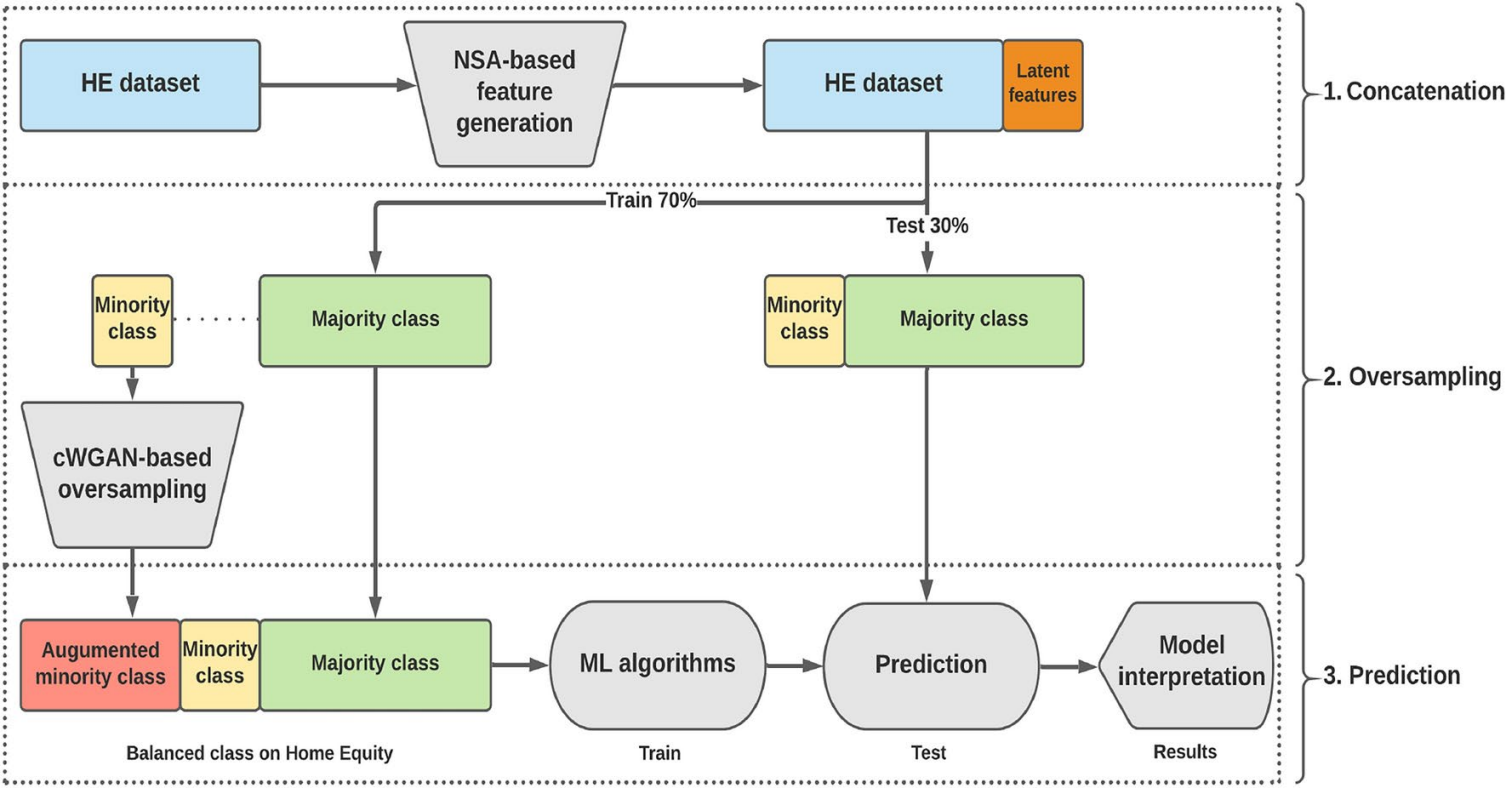
The system architecture of NOTE.

### Prediction

Following the oversampling of the minority class using cWGAN, NOTE proceeds with predictions using machine learning (ML) models and explanations provided by ‘TreeExplainer’ and ‘LinearExplainer’. Figure 5 illustrates the system architecture of NOTE. The five ML classifiers utilized for performance comparison, including tree-based models, are as follows:

Logistic regression (LR) has long been a standard model for binary classification tasks^40^. Decision tree (DT) classifiers operate by recursively partitioning the dataset based on informational criteria for classification purposes^41^. Extra Trees (ET) is an ensemble method that aggregates the outcomes of multiple decision tree classifiers^42^, similar to the Random Forest (RF) approach, which also employs an ensemble of decision tree classifiers^43^. Although ET and RF are conceptually alike, they differ in the methodology used to construct the decision trees within the ensemble. Gradient Boosting (GB) is a technique that enhances classification performance by combining multiple weak classifiers into a robust model^44^. In this study, DTs serve as the base learners for the GB implementation.

Tree-based machine learning ensemble classifiers, such as Extra Trees (ET), Random Forest (RF), and Gradient Boosting (GB), are among the most widely used non-linear predictive models^45,46^. These models are frequently employed in domains where predictions must be both accurate and interpretable, such as in medicine and finance^47^. In these fields, it is essential to balance predictive accuracy with explainability, which refers to the ability to understand how the classifiers utilize input features to generate their predictions^46^.

Since logistic regression (LR) employs a logistic function, its coefficients are easily interpretable. However, it uses a linear decision boundary, which means it ignores complex interactions between variables. In contrast, tree-based algorithms such as Random Forest (RF) and Gradient Boosting (GB) can model complex and non-linear decision boundaries. This complexity, however, makes tree-based models more difficult to interpret in terms of their predictions.

Although decision trees (DT) can be interpreted through their decision paths, the construction of multiple trees in tree-based ensemble models, such as Random Forest and Gradient Boosting, makes their predictions less interpretable.

## Results

This section assesses both the generative and predictive performance of NOTE.

### The generative performance

Prior to comparing classification performance achieved through oversampling among various methods-none, NOTE, ADS-GAN^14^, DeepSMOTE^15^, and cWGAN as benchmarks^12^-it is essential to evaluate the distribution of synthetic data to assess the generative performance of NOTE. Table 2 provides a comparison of the number of instances per class in the original imbalanced dataset and the balanced dataset after oversampling the minority class. To analyze the generative performance of NOTE, the convergence of the cWGAN model was evaluated using a variety of performance metrics, including generator and discriminator losses, Wasserstein distance, and gradient penalty. These metrics provide insights into the stability and convergence of the training process, which are essential for ensuring the generation of high-quality synthetic data. The generative performance of the NOTE framework was evaluated using the HE dataset.

**Table 2:** Class distribution following minority oversampling using NOTE on HE and GMSC datasets.

HE	# Original	# Generated	Total	GMSC	# Original	# Generated	Total
# Not defaulted (0)	4,771	0	4,771	# Not defaulted (0)	139,974	0	139,974
# Defaulted (1)	1,189	3,582	4,771	# Defaulted (1)	10,026	129,948	139,974
**Total**	5,960	3,582	9,542	**Total**	150,000	129,948	279,948

**Figure 6:**
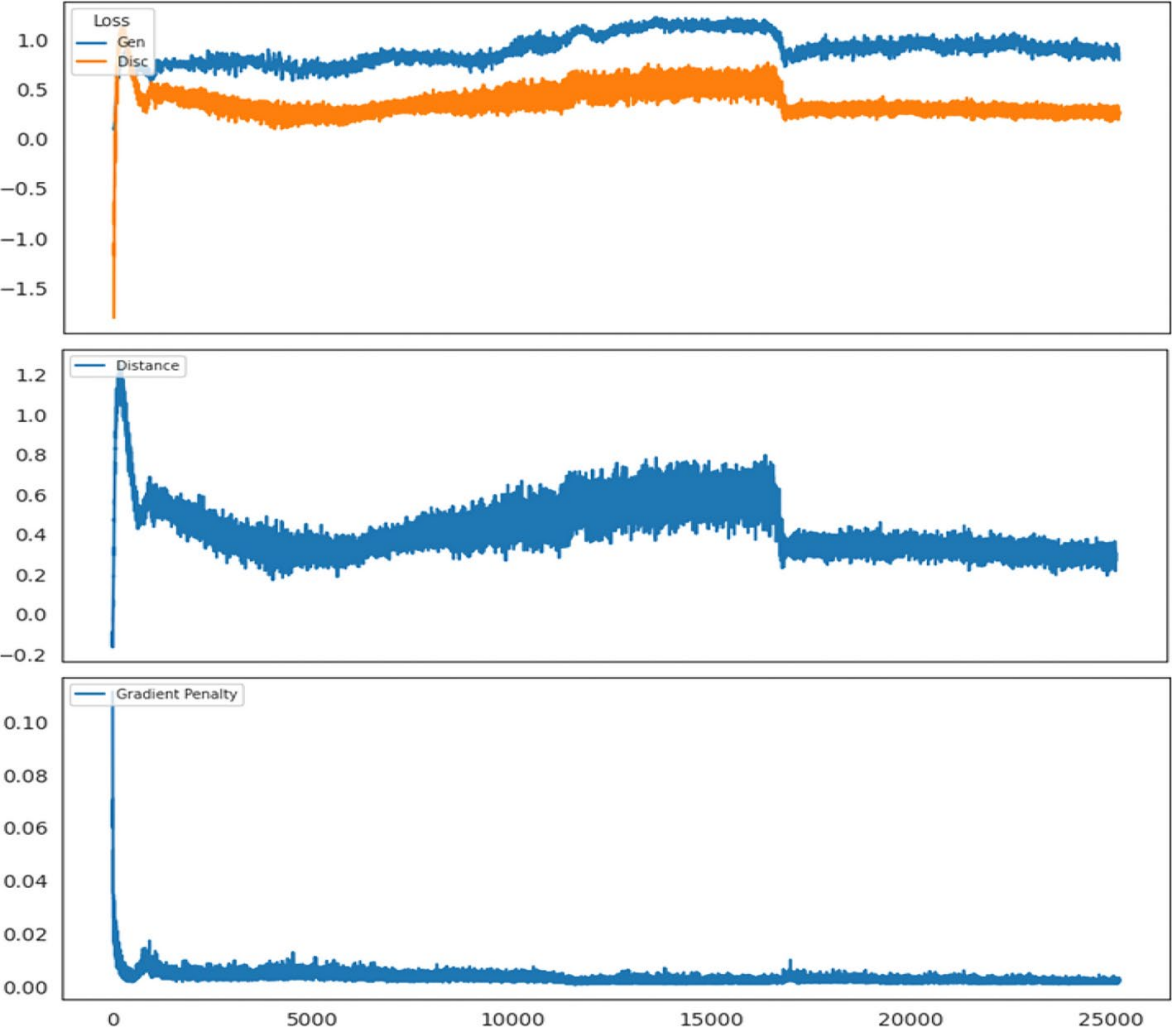
cWGAN-GP training metrics: generator and discriminator loss(top), Wasserstein distance(middle), and gradient penalty(bottom).

As illustrated in top of Fig. 6, the generator (blue line) and discriminator (orange line) losses exhibit stabilizing behavior over time. Initially, both losses fluctuate, reflecting the adversarial nature of GAN training, wherein the generator and discriminator attempt to improve relative to one another. Over time, the generator’s loss stabilizes around a consistent value, indicating that it has reached a stable point, producing data that the discriminator evaluates consistently. Similarly, the discriminator loss stabilizes after an initial decline, suggesting that its ability to distinguish between real and synthetic data is no longer improving significantly. The consistent gap between the generator and discriminator losses indicates a balanced adversarial process, which is commonly interpreted as a sign of convergence and equilibrium.

The Wasserstein distance as shown in the middle of Fig. 6 is a critical metric for evaluating cWGAN performance. The generator’s objective is to minimize the Wasserstein distance between the real and synthetic data distributions. Initially, this distance fluctuates but gradually decreases and stabilizes, signifying that the generator is learning to approximate the real data distribution effectively. A stabilized and reduced Wasserstein distance is a strong indication that the model has successfully converged.

The gradient penalty as shown in the bottom of Fig. 6 also demonstrates an initial high value that decreases and stabilizes over time. This reflects the model’s effective enforcement of the Lipschitz continuity condition, which is critical for maintaining stable gradients during training. The stabilization and low value of the gradient penalty further corroborate the model’s convergence and controlled gradient behavior throughout training.

In summary, the combination of stabilized generator and discriminator losses, a decreasing and stable Wasserstein distance, and a well-controlled gradient penalty strongly indicates that the cWGAN-GP model has successfully converged.

**Figure 7:**
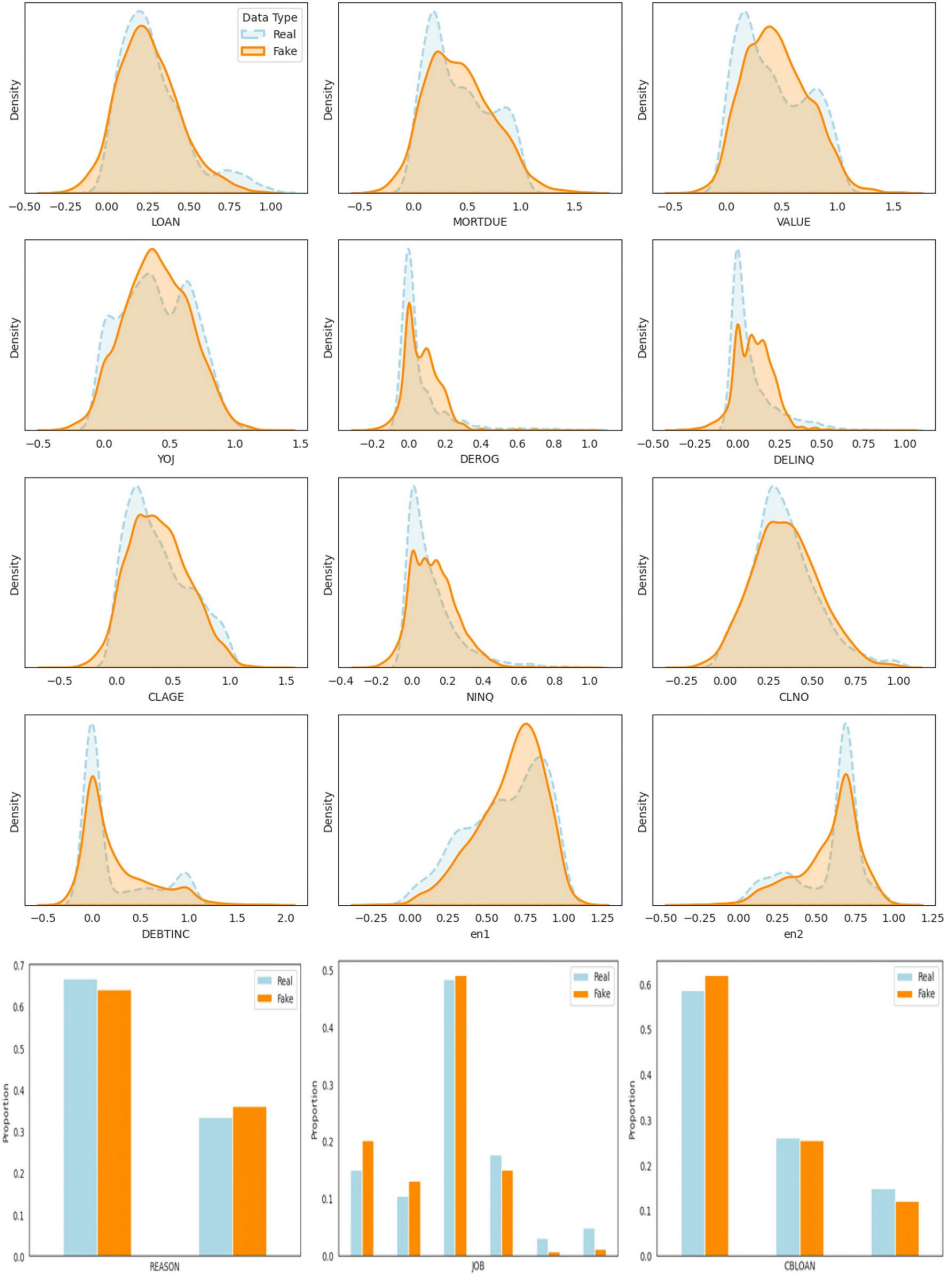
Comparison of real and synthetic data for numerical and categorical features in the HE dataset, with latent representations ‘en1’ and ‘en2’ extracted through NSA.

Beyond assessing model convergence, it is crucial to evaluate the quality of the generated synthetic data in order to comprehensively assess the overall performance of the cWGAN model. Figure 7 presents a series of density and bar plots that compare the distributions and frequencies of real and synthetic data across various numerical and categorical variables. These visualizations offer an insightful representation of how effectively the synthetic data generated by the cWGAN approximates the distributions and frequencies of key features in the original real dataset.

For the majority of variables, such as LOAN, MORTDUE, VALUE, CLNO, and DEBTINC, the synthetic data exhibits a strong alignment with the real data distribution, particularly around the median values. This suggests that the cWGAN model has successfully captured the overall structure and range of these features, demonstrating robust performance in generating realistic synthetic data.

However, the model faces difficulties in accurately replicating the distributions of more skewed or imbalanced features, such as DEROG, DELINQ, and NINQ, where the real data exhibits a high concentration of values near zero. These discrepancies indicate that further refinement of the model is required to more effectively capture tail behaviors and the distributions associated with rare occurrences.

In summary, while the cWGAN model demonstrates strong performance across the majority of continuous features, further tuning may be necessary to address the challenges associated with generating realistic data for rare or extreme values.

Continuing the analysis with the bar plots in Fig. 7, which compares the distributions of real and synthetic data for categorical variables such as REASON, JOB, and CBLOAN, similar patterns are observed in the cWGAN model’s performance when generating discrete features.

For REASON, the synthetic data exhibits a high level of consistency with the real data, particularly within the primary categories. The model successfully replicates the proportional distribution of the two main reasons for obtaining loans, with only minor deviations observed in the less frequent categories. This indicates the model’s capacity to capture the overall distribution of categorical features, particularly where the categories are relatively balanced.

In the case of JOB, the cWGAN model similarly performs well in generating realistic data. The distribution of synthetic data closely aligns with the real data for the major job categories, demonstrating the model’s effectiveness in replicating the proportions of dominant categories. However, as with the continuous variables, minor discrepancies are evident in the less frequent job categories, indicating that the model encounters some difficulty in generating data for underrepresented categories. This reflects the broader challenge of accurately replicating the distributions of rare or skewed continuous variables.

For CBLOAN, the synthetic data once again closely aligns with the real data, particularly in the more common categories. The model effectively captures the primary structure of loan statuses, as reflected by the similar proportions of real and synthetic data in the major categories. However, minor discrepancies in the less frequent categories indicate that, while the model performs well overall, further refinement may be necessary to improve accuracy in generating data for smaller categories.

In summary, Fig. 7 illustrates that the cWGAN model exhibits strong performance in generating realistic synthetic data across most continuous and categorical variables. Although the model effectively captures the structure of core features, additional tuning may be necessary to improve its capacity to replicate the distributions of rare or highly skewed variables. The minor discrepancies observed in less frequent categories and imbalanced distributions indicate that further refinement is required to enhance the model’s ability to generate high-fidelity data across all variable types.

After the cWGAN model successfully learns the distribution of the real data, it generates 3,582 synthetic bad credit samples to address the class imbalance, given that the original dataset contains 1,189 bad credit samples in the minority class. These synthetic samples, which closely mirror the distribution of the real dataset as previously discussed, are incorporated into the minority class. Consequently, the total number of bad credit samples in the HE dataset is increased to 4,771, thereby equaling the number of good credit samples, as shown in Table 2.

In a similar manner, for the GMSC dataset, where the initial distribution consists of 139,974 non-defaulted (good credit) samples and 10,026 defaulted (bad credit) samples, the cWGAN model generates an additional 129,948 synthetic defaulted samples. This augmentation results in a total of 139,974 defaulted samples, effectively balancing the dataset by ensuring that both defaulted and non-defaulted classes contain 139,974 samples. The final dataset, therefore, comprises 279,948 samples in total, providing a balanced class distribution for subsequent prediction model training.

### The predictive performance

To evaluate the proposed model NOTE against established benchmarks^12^ for oversampling and rSVD for feature extraction, a comprehensive analysis using various classifiers was conducted. Specifically, tree-based models including Decision Trees (DT), Extra Trees (ET), Random Forests (RF), and Gradient Boosting (GB), along with Logistic Regression (LR), were applied within the domain of credit scoring. These classifiers were also trained on the original imbalanced dataset to rigorously assess the effectiveness of oversampling the minority class and capturing latent features that represent non-linearity. This approach ensures a thorough comparison of the models’ abilities to handle class imbalance and model complex data patterns.

Given that Engelmann and Lessmann^12^ recommended hyperparameter tuning as an avenue for future work, highlighting its potential for enhancing model performance, we have validated the robustness of the NOTE framework by implementing grid search for hyperparameter optimization. This process was carried out within a 5-fold cross-validation, ensuring a comprehensive search across the parameter space to identify the optimal hyperparameters.

**Table 3:** AUC comparison after hyperparameter optimisation* between non-resampling and oversampling methods (NOTE, ADSGAN, DeepSMOTE) with two latent representations on the HE and GMSC datasets (benchmarks^12^ in brackets, best AUC highlighted in bold).

HE	None	NOTE	ADSGAN	DeepSMOTE	GMSC	None	NOTE	ADSGAN	DeepSMOTE
LR	0.8521 (0.7738)	**0.9595** (0.7554)	0.8395	0.9535	LR	0.7984 (0.6981)	**0.9750** (0.7449)	0.7748	0.8146
DT	0.7844 (0.7867)	**0.9546** (0.7935)	0.8112	0.9443	DT	0.5814 (0.6084)	**0.9648** (0.6090)	0.9546	0.8312
ET	0.9706 (N/A)	**0.9913** (N/A)	0.9411	0.9608	ET	0.7926 (N/A)	**0.9831** (N/A)	0.9672	0.8588
RF	0.9550 (0.9733)	**0.9891** (0.9761)	0.8708	0.9571	RF	0.8181 (0.8415)	**0.9837** (0.8412)	0.9766	0.8530
GB	0.9336 (0.9213)	**0.9891** (0.9119)	0.8525	0.9602	GB	0.8153 (0.8330)	**0.9834** (0.8304)	0.9754	0.8544

* Search space for hyperparameters:

LR: penalty={’none’, ’L1’, ’L2’, ’elasticnet’}, inverse penalty coefficient C=loguniform(1e-5, 100), solver={’newton-cg’, ’lbfgs’, ’liblinear’}

DT: max_features={’auto’, ’sqrt’}, max_depth=[1, 20], min_samples_split={1, 2, 5, 10}, min_samples_leaf={1, 2, 4, 8}

ET: n_estimators=[100, 1000], max_features={’auto’, ’sqrt’}, max_depth=[1, 20], min_samples_split={1, 2, 5, 10}, min_samples_leaf={1, 2, 4, 8}, bootstrap={’True’, ’False’}

RF: n_estimators=[100, 1000], max_features={’auto’, ’sqrt’}, max_depth=[1, 20], min_samples_split={1, 2, 5, 10}, min_samples_leaf={1, 2, 4, 8}, bootstrap={’True’, ’False’}

GB: n_estimators=[100, 1000], learning_rate={0.01, 0.1, 0.5}, max_depth=[1, 20], min_samples_split={1, 2, 5, 10}, min_samples_leaf={1, 2, 4, 8}, subsample={0.8, 1.0}

N/A = No results available in benchmarks^12^

**Figure 8:**
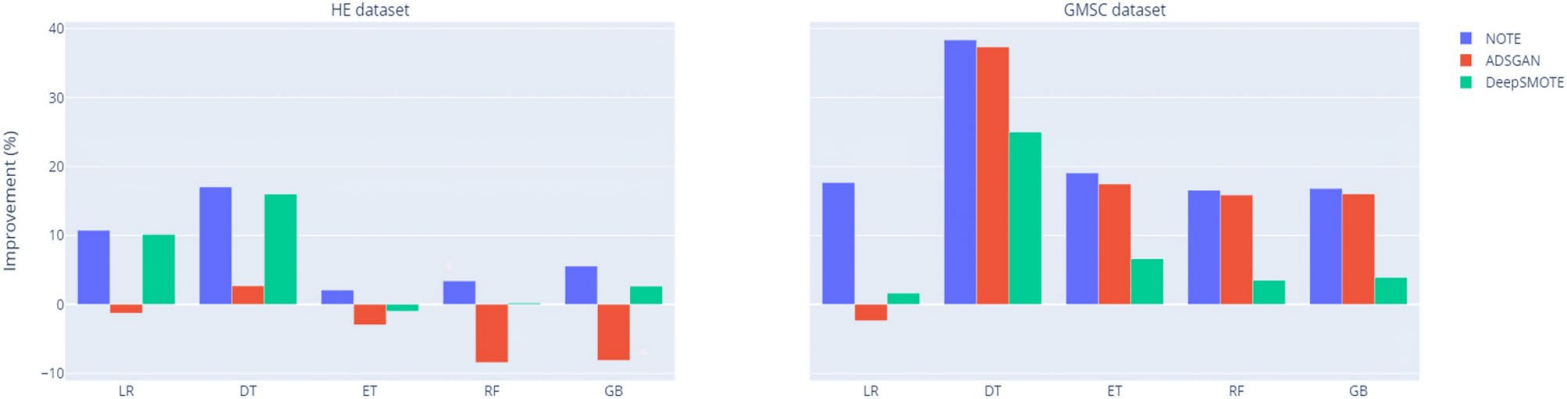
AUC improvement of classification models by oversampling methods against non-resampling.

Following the selection of the best hyperparameters, we further evaluated model performance using 10-fold cross-validation, with Area Under the Curve (AUC) as the primary metric. AUC is widely recognized as a robust metric for evaluating classification performance, particularly in imbalanced datasets^48,49^, ensuring a balanced assessment of the model’s predictive capabilities across different classes.

Additionally, to address concerns about overfitting-particularly relevant for tree-based models and oversampling techniques-all tree-based classifiers within the NOTE framework were pruned to prevent overfitting and enhance generalizability. This precaution aligns with findings from Engelmann and Lessmann^12^, where benchmark results were reported without hyperparameter optimization, potentially limiting model robustness.

Our evaluation compares the baseline performance of the original imbalanced dataset with NOTE, as well as recent oversampling methods like ADS-GAN^14^ and DeepSMOTE^15^, to demonstrate the framework’s effectiveness. This benchmark analysis, alongside previous findings in the credit scoring literature^12^, is utilized to compare the performance of NOTE with established benchmarks. Specifically, it evaluates NOTE’s capacity to extract latent features and enhance classification performance in non-linear and imbalanced datasets, demonstrating its effectiveness relative to prior research in the field.

The results presented in Table 3 and Fig. 8 provide a comprehensive comparison of classification performance across multiple resampling techniques, including NOTE, ADSGAN, and DeepSMOTE, in relation to the baseline non-resampling approach (None). The AUC values clearly demonstrate the superior performance of the NOTE framework across both the HE and GMSC datasets, with notable improvements evident in all classifiers examined.

In both datasets, the NOTE framework consistently outperforms the most recent oversampling methods, ADSGAN and DeepSMOTE, as well as the non-resampling baseline. For instance, in the HE dataset, the AUC for LR using NOTE achieves 0.9595, representing a substantial improvement over ADSGAN (0.8395), DeepSMOTE (0.9535), and the baseline (0.8521). Similarly, the DT classifier with NOTE records an AUC of 0.9546, surpassing ADSGAN (0.8112) and DeepSMOTE (0.9443).

This trend of superior performance is further corroborated in the GMSC dataset, where the NOTE framework consistently achieves the highest AUC values across multiple classifiers. For example, the RF attains an AUC of 0.9837 with NOTE, compared to 0.9766 with ADSGAN and 0.8530 with DeepSMOTE. Similar patterns of improvement are observed in other classifiers, including ET and GB, where NOTE delivers higher AUCs, indicating enhanced robustness and classification accuracy.

**Table 4:** AUC comparison after hyperparameter optimisation* between NOTE and cWGAN with rSVD on the HE and GMSC datasets. Benchmarks^12^ for cWGAN oversampling without extraction (best AUC highlighted in bold).

HE	NOTE	rSVD	rSVD and NSA	Engelmann and Lessmann^12^	GMSC	NOTE	rSVD	rSVD and NSA	Engelmann and Lessmann^12^
LR	**0.9595**	0.8152	0.9257	0.7554	LR	**0.9750**	0.9562	0.9643	0.7449
DT	**0.9546**	0.8682	0.9227	0.7935	DT	**0.9648**	0.9642	0.9641	0.6090
ET	**0.9913**	0.9777	0.9804	N/A	ET	**0.9831**	0.9735	0.9776	N/A
RF	**0.9891**	0.9685	0.9775	0.9761	RF	**0.9837**	0.9806	0.9816	0.8412
GB	**0.9891**	0.9733	0.9803	0.9119	GB	**0.9834**	0.9822	0.9829	0.8304

* Search space for hyperparameters is the same as Table 3

**Figure 9:**
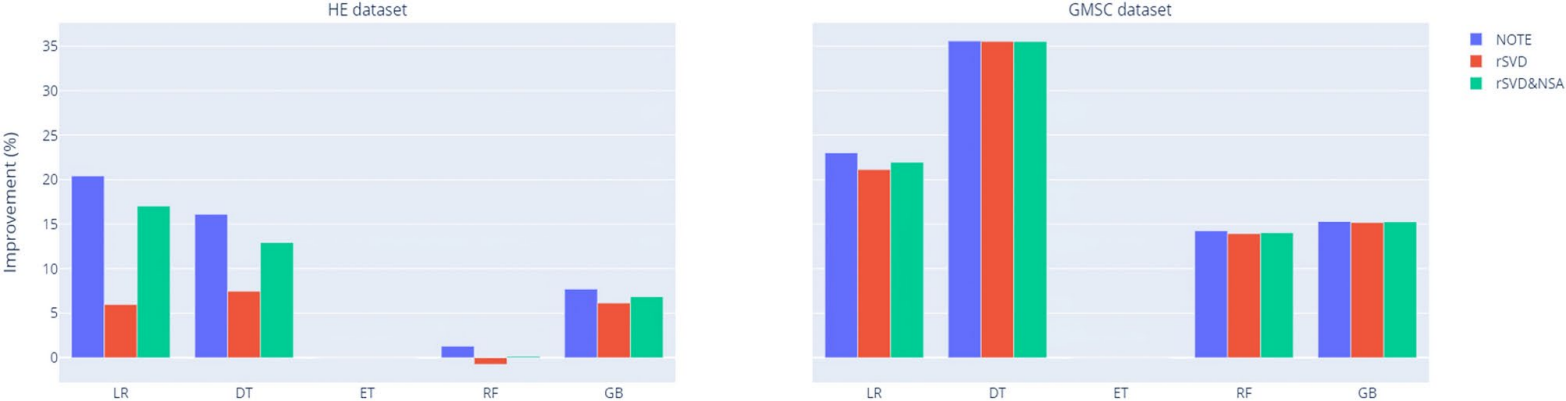
AUC improvement of classification models by extracting and denoising methods against benchmarks^12^ (AUC of ET: N/A).

These findings clearly establish that the NOTE framework not only surpasses the non-resampling baseline, but also exceeds the performance of contemporary oversampling techniques. The widespread improvements across various classifiers, particularly in tree-based ensemble methods such as ET, RF, and GB, underscore the robustness of the NOTE framework in addressing non-linear and imbalanced datasets. Overall, these results demonstrate the practical effectiveness of the NOTE framework in improving classification accuracy, reinforcing its superiority through thorough experimental validation.

Furthermore, the robustness of extracting latent vectors using NSA is assessed by comparing it to noise reduction achieved through rSVD. Singular Value Decomposition (SVD), a prevalent method for matrix decomposition, is widely utilized for dimensionality reduction, data analysis, and data compression^50^. This comparative evaluation aims to determine the efficacy of NSA in maintaining data integrity and capturing essential features.

Although SVD is computationally intensive, randomized SVD (rSVD) has been introduced to mitigate these costs, facilitating scalable matrix transformations while effectively capturing latent information within datasets^51^. Given that Engelmann and Lessmann^12^ identified non-linearity in both the HE and GMSC datasets, the rSVD method is also applicable for data reconstruction. The proposed NOTE model is compared with cWGAN-based oversampling techniques, utilizing rSVD, to determine which method more effectively enhances cWGAN’s performance in generating realistic synthetic distributions and improves classifier accuracy in predicting classifications.

Table 4 and Fig. 9 provide a comparative analysis of AUC performance across five classifiers-LR, DT, ET, RF, GB-evaluating different methods such as NOTE, rSVD, rSVD and NSA, alongside the benchmark results from Engelmann and Lessmann^12^. Notably, the results demonstrate that the NOTE framework consistently outperforms the other methods, particularly when applied to tree-based ensemble classifiers, highlighting its robustness in handling non-linear and imbalanced datasets.

In the case of LR, the NOTE framework achieves AUC scores of 0.9595 for the HE dataset and 0.9750 for the GMSC dataset, substantially surpassing the benchmark scores (0.7554 for HE and 0.7449 for GMSC). While rSVD and rSVD with NSA show improvements over the benchmarks, they do not reach the same level of performance as NOTE, demonstrating its superior ability to capture important latent information and reduce noise.

Similarly, with DT, the NOTE framework yields higher AUC values of 0.9546 for HE and 0.9648 for GMSC, significantly outperforming the benchmark results (0.7935 for HE and 0.6090 for GMSC). Although rSVD and rSVD with NSA show competitive results, NOTE’s ability to handle non-linear structures is further emphasized, aligning with the conclusions of Engelmann and Lessmann^12^, who identified the non-linear nature of the HE dataset.

For ET, the NOTE framework achieves its highest AUC scores, recording 0.9913 for HE and 0.9831 for GMSC. While rSVD and rSVD with NSA also show strong performance (0.9804 for HE and 0.9776 for GMSC), they still fall short of NOTE’s efficacy, underscoring its strength in extracting meaningful latent information from non-linear data.

When applied to RF, the NOTE framework continues to outperform the other methods, achieving AUC values of 0.9891 for HE and 0.9837 for GMSC. Although rSVD and rSVD with NSA are competitive, NOTE consistently delivers the best results, reinforcing its effectiveness in denoising and improving classification accuracy.

In GB, NOTE also demonstrates its superiority, with AUC values of 0.9891 for HE and 0.9834 for GMSC. While rSVD and rSVD with NSA provide decent improvements over the benchmark, NOTE remains the best performer, showcasing its capability to enhance classification performance, particularly in non-linear models.

Consequently, the results validate that the proposed NOTE framework, particularly when used with tree-based ensemble classifiers, significantly enhances classification performance in non-linear and imbalanced datasets. This improvement is achieved by leveraging NSA for latent vector extraction and rSVD for noise reduction. The findings align with the recommendations of Engelmann and Lessmann^12^, who emphasized the efficacy of tree-based models in such contexts. Moreover, the benchmarks reported by Engelmann and Lessmann were surpassed through the application of NSA and rSVD, further underscoring the robustness of the NOTE framework in improving classifier accuracy.

### Statistical assessment of NOTE performance

The Friedman test was utilized to assess the performance of the NOTE framework due to its effectiveness in analyzing repeated measures across multiple models. As a non-parametric test, the Friedman test is specifically designed for situations where repeated measures are applied to the same datasets^52^, making it well-suited for our study. This test evaluates whether there are statistically significant differences in the performance of different methods. Its application was particularly relevant here, as it enabled us to compare the ranks of AUC values across the different techniques. By doing so, the Friedman test provided a robust statistical basis for determining whether the improvements observed with the NOTE framework are significant in comparison to the alternative approaches.

**Table 5:** Friedman test results for HE and GMSC datasets (p < 0.05 highlighted in bold).

Table 3	Statistic (Chi-Square)	p-value	Significance	Table 4	Statistic (Chi-Square)	p-value	Significance
HE	15.44	**0.0039**	Significant	HE	9.92	**0.0192**	Significant
GMSC	11.40	**0.0224**	Significant	GMSC	9.90	**0.0194**	Significant

The results presented in Table 5 provide the Friedman test outcomes for two sets of comparisons. The left side of the table assesses the performance differences between NOTE, ADSGAN, and DeepSMOTE, using non-oversampling (None) as the baseline. In contrast, the right side compares NOTE, rSVD, rSVD combined with NSA, and the benchmark method proposed by Engelmann and Lessmann^12^. Across both the HE and GMSC datasets, statistically significant differences are observed, as evidenced by the chi-square statistics and p-values, all of which are below the 0.05 threshold.

In the comparison between NOTE, ADSGAN, and DeepSMOTE (left side), the HE dataset yields a chi-square statistic of 15.44 with a p-value of 0.0039, while the GMSC dataset reports a chi-square statistic of 11.40 and a p-value of 0.0224. These findings indicate that the differences in classification performance among the oversampling methods are statistically significant. Given NOTE’s consistent superiority in AUC across the classifiers, it can be concluded that NOTE demonstrates substantial improvement over both ADSGAN and DeepSMOTE.

On the right side, comparing NOTE with rSVD, rSVD combined with NSA, and the Engelmann and Lessmann benchmark^12^, the HE dataset produces a chi-square statistic of 9.92 with a p-value of 0.0192, while the GMSC dataset yields a chi-square statistic of 9.90 and a p-value of 0.0194. These significant p-values suggest that the performance differences between the methods are also non-random. Once again, the NOTE framework outperforms both rSVD and rSVD combined with NSA, underscoring its effectiveness in feature extraction.

Overall, the Friedman test results provide robust statistical evidence that NOTE consistently outperforms alternative methods in both oversampling and feature extraction, further reinforcing its effectiveness in improving classification performance across diverse datasets.

## Discussion

In this study, we implemented the non-parametric stacked autoencoder (NSA) and conditional Wasserstein GAN (cWGAN) as two distinct models, which were trained sequentially. This approach was selected due to the specific and independent functions of each model: the NSA was utilized for latent feature extraction, while the cWGAN was employed to tackle the challenges associated with generating synthetic data for imbalanced datasets. The sequential nature of this design allowed for a clear and focused evaluation of each model’s individual performance. However, this methodology presents a limitation in that NSA does not adapt in real-time to the knowledge gained by cWGAN during the generation process. As a result, errors generated by the NSA could propagate to the cWGAN, which could potentially restrict the opportunity for simultaneous optimization and deeper integration between feature extraction and data generation. This may result in suboptimal performance compared to an integrated model where the two components interact more dynamically.

Moreover, while the results from the Home Equity and Give Me Some Credit datasets are promising, we acknowledge that limiting the evaluation of the NOTE framework to only two datasets represents a constraint of this study. This limitation has been addressed by highlighting the need for further validation across a broader spectrum of credit scoring datasets to ensure greater generalizability.

Various model-agnostic XAI techniques, such as SHAP (SHapley Additive exPlanations)^46^, LIME (Local Interpretable Model-agnostic Explanations)^53^, and PDP (Partial Dependence Plots)^54^, hold significant potential for enhancing the interpretability of models like NOTE. In particular, a study by^46^ emphasizes the explainability of tree-based models by analyzing feature contributions. An earlier study by^13^ introduced TreeExplainer, which employs SHAP, a method grounded in Shapley values from game theory^55^. SHAP facilitates the interpretation of machine learning predictions by estimating the contribution of individual features, thus providing insights into both the global and local effects of features on the model’s predictions. SHAP values offer a transparent and interpretable representation of feature attributions.

As preliminary experiments within the NOTE framework, SHAP was utilized to explore the interpretability of the model’s predictions. The interpretability analysis generated by SHAP offers a detailed examination of the model’s decision-making process, combining local and global interpretability techniques to enhance the understanding of both individual predictions and overall feature importance. SHAP provides a multi-dimensional approach to model interpretation, balancing explanations at both the local and global levels.

At the local level, it enables a detailed examination of individual predictions, revealing how specific features contribute to each prediction. For instance, in a non-default prediction of 0.22 in the GMSC dataset, features such as ‘age (34)’ and ‘RevolvingUtilizationOfUnsecuredLines (0.799)’ increase the likelihood of default by 0.03 and 0.1, respectively, while ‘CombinedDefaulted (0)’ and ‘NumberOfTimes30-59DaysPastDueNotWorse (0)’ reduce it by 0.2 and 0.12. Conversely, for a default prediction of 0.76, ‘CombinedDefaulted (1)’ and ‘RevolvingUtilizationOfUnsecuredLines (0.796)’ increase the default likelihood by 0.18 and 0.09, while ‘NumberOfDependents (0)’ and ‘DebtRatio (0.038)’ reduce it by 0.05 and 0.01.

At the global level, the analysis identifies ‘CombinedDefaulted’ as the feature with the highest average SHAP value of 0.31 across the dataset, highlighting its dominant influence on the model’s predictions. Other significant features, such as ‘RevolvingUtilizationOfUnsecuredLines’ and ‘NumberOfTimes30-59DaysPastDueNotWorse’, have mean SHAP values of 0.22 and 0.21, respectively. This comprehensive analysis is particularly beneficial in high-stakes decision-making environments, such as credit risk assessment, where both specific cases and broader trends must be considered to ensure transparency and reliability in AI systems. By analyzing both individual cases and overall patterns, SHAP provides valuable insights into the model’s behavior, contributing to more transparent and interpretable machine learning processes.

The focus on interpretability within the NOTE framework strikes an effective balance between model complexity and the generation of actionable insights, making the predictions more accessible and comprehensible to stakeholders. This approach aligns with recent studies, such as^56^, which highlight the importance of hypothesis-driven decision-making. By adopting this perspective, the NOTE framework maximizes the predictive power of advanced models while ensuring the provision of meaningful explanations for its outputs. This balance between predictive performance and interpretability strengthens NOTE’s effectiveness as a predictive tool and enhances its practical application in credit scoring.

This analysis is consistent with recent research advocating for a shift towards hypothesis-driven decision-making in XAI^56^. The integration of local evidence with global insights aligns with the evaluative AI framework proposed in that study, where decision-makers actively participate in hypothesis generation and testing, rather than merely receiving AI-generated recommendations. By examining both supporting and opposing evidence for specific decisions, evaluative AI helps mitigate the risks of over-reliance on machine-generated predictions and fosters a deeper understanding of decision-making processes. SHAP exemplifies this approach by allowing users to explore both localized and global explanations, thereby encouraging an evidence-based engagement with the model’s reasoning.

A promising direction for future research would be to explore the integration of the NSA and cWGAN into a unified model. In such a framework, the outputs from the coding layer of the NSA could be directly fed into the cWGAN, allowing both components to be trained simultaneously within a single step. This integration would enable the NSA to adjust dynamically based on how the cWGAN models non-linear structures within the data, thereby fostering a more cohesive and interactive learning process. The proposed joint optimization of feature extraction and data generation could lead to significant improvements in overall model performance by allowing both models to benefit from each other’s feedback during training. We intend to investigate the development and implementation of this integrated model in future research efforts, as it holds the potential to address the limitations of the current sequential approach and further advance the efficacy of synthetic data generation for imbalanced datasets.

In addition to the proposed model integration, we also recognize the importance of further validating the generalizability of the NOTE framework. As suggested, future work will involve extending the evaluation to additional public credit scoring datasets, such as the German, Australian, Taiwanese, and Polish datasets from the UCI Machine Learning Repository. This extended evaluation will allow for a more thorough assessment of the framework’s robustness and applicability across diverse contexts. By incorporating a wider range of datasets, we aim to enhance our understanding of NOTE’s effectiveness and ensure its adaptability to various credit scoring scenarios.

Furthermore, we acknowledge that improving the generative performance of the NOTE framework through more advanced techniques is an important direction for future research. As noted, while the current use of an Autoencoder (AE) facilitates feature extraction, the introduction of a Variational Autoencoder (VAE) could address potential limitations related to the continuity of the latent space, ensuring that generated data more accurately reflects the original distribution. Incorporating VAE into the NOTE framework may enhance the quality and consistency of synthetic data, particularly in challenging imbalanced datasets. We plan to explore the integration of VAE in future work, as this approach holds the potential to refine the generative process and further improve the framework’s overall performance. By addressing the limitations of the standard AE approach, this adjustment would provide more robust data generation and greater alignment with real data distributions.

## Conclusion

This paper presents a novel non-parametric oversampling technique, termed NOTE, specifically designed to address the challenges posed by imbalanced classes in non-linear credit scoring datasets. The evaluation involved a comprehensive comparison of three oversampling methods, two feature extraction techniques, and five distinct classification algorithms, benchmarked against established standards.

The findings demonstrate that NOTE effectively generated minority class samples by leveraging the strengths of NSA, cGAN, and WGAN. As a result, the proposed framework significantly enhanced classification performance compared to the standard LR model on non-linear imbalanced credit scoring datasets, surpassing previous studies utilizing cWGAN-based oversampling methods. Additionally, NOTE’s explainability, achieved through both local and global interpretations via TreeExplainer and LinearExplainer, alongside its robustness in managing non-linear datasets, underscores its practical value in credit scoring applications.

Future research could explore the integration of deep learning techniques with the tree-based ensemble models utilized in NOTE, aiming to develop a hybrid approach that further enhances robustness in capturing non-linear patterns. Moreover, validating NOTE’s efficacy on more severely imbalanced or entirely imbalanced non-linear datasets across various domains would extend its generalizability and broader applicability.
